# In Silico Analysis of Tat Exons to Increase the Efficacy of a Nef-Tat-based HIV-1 Vaccine Candidate

**DOI:** 10.5812/ijpr-162036

**Published:** 2025-09-13

**Authors:** Leila Sadeghi, Fatemeh Heidarnejad, Azam Bolhassani, Elham Mohit, Parisa Moradi Pordanjani

**Affiliations:** 1 Department of Hepatitis, AIDS and Blood-Borne Diseases, Pasteur Institute of Iran, Tehran, Iran; 2 Department of Pharmaceutical Biotechnology, School of Pharmacy, Shahid Beheshti University of Medical Sciences, Tehran, Iran

**Keywords:** HIV-1, Nef, Tat, Immunoinformatics Analysis, Vaccine

## Abstract

**Background:**

The global human immunodeficiency virus HIV/AIDS pandemic persists without complete eradication. Developing a safe and effective vaccine remains the most promising approach, but ongoing clinical trials have been unsuccessful due to the vaccines' inability to stimulate robust immunity.

**Objectives:**

The present research endeavor proposes an innovative therapeutic vaccine by employing immunoinformatics strategies. Herein, we aimed to compare the efficiency of the whole sequence of Tat^(exons 1 + 2)^ with its first exon^(exon 1)^ in a fusion vaccine construct harboring the whole sequence of the Nef protein [i.e., Nef-Tat^(exons 1 + 2)^ and Nef-Tat^(exon 1)^ fusion proteins] using in silico studies.

**Methods:**

First, the secondary structures of both fusion proteins were predicted. Then, 3D models of the constructs were refined, and their physicochemical properties were determined. After analysis of toxicity, allergenicity, and antigenicity of constructs, the formation of ligand (constructs)-receptor (TLR-2 to TLR-5, and TLR-7 to TLR-9) complexes was examined using the ClusPro and HDOCK servers, and the highest scores of docking analysis were used for molecular dynamics (MDs) simulation. Finally, the JCat server was applied for codon optimization.

**Results:**

Our results indicated that both protein constructs were antigenic, non-allergenic, and capable of eliciting adaptive immune responses. The average values of radius of gyration (Rg) for Nef-Tat^(exon 1)^ and Nef-Tat^(exons 1+2)^ with TLR-4 were 1.74 and 1.90, respectively. Therefore, both constructs were stable. Moreover, the Nef-Tat^(exon 1)^ construct could significantly activate both T- and B-cells as compared to the Nef-Tat^(exons 1+2)^. Indeed, inclusion of the second exon of Tat^(exon 2)^ did not enhance the immunogenicity of the Nef protein.

**Conclusions:**

Generally, immunoinformatics studies showed the importance of Tat exon 1 in HIV-1 fusion vaccine design.

## 1. Background

Millions of people are affected by human immunodeficiency virus (HIV) worldwide (1). To date, the only available treatment for infected individuals relies on antiretroviral therapy (ART). However, due to the increasing numbers of infected people and the costs of ART treatment (2), therapeutic vaccines are being explored to control HIV replication (3). Subunit vaccines such as UVAX-1107 and UVAX-1197, which are protein-based HIV vaccine candidates (4), can reduce the likelihood of adverse reactions compared to whole microorganism vaccines. To successfully combat HIV infection, it is essential to identify an efficient antigen (5).

An ideal HIV vaccine should elicit neutralizing antibodies and also early and effective T-cell responses to combat the large diversity of HIV variants and suppress initial viremia, respectively (2). The HIV-1 Tat and Nef, expressed early with conserved immunogenic epitopes, play key roles in the viral lifecycle and AIDS progression and are ideal vaccine targets. Their induced immune responses correlate with slower disease progression (6). Various vaccine studies using Nef and Tat antigens from HIV-1 or simian immunodeficiency virus (SIV) in different forms (protein, peptide, DNA, vectors) have indicated their safety and ability to induce immune responses in animals and humans. The HIV-1 Tat, encoded by two exons, has a conserved exon 1 important for transactivation, while exon 2 enhances HIV replication in T-cells. The first exon of the Tat protein includes several functional regions: The N-terminal proline-rich region, the cysteine-rich region, the core region, and the basic region for nuclear localization and binding to the HIV LTR TAR RNAs. The second exon of Tat contributes to the virus's infectivity, expression, and replication in T-cells and in monocyte-derived dendritic cells (DCs) (7). It was reported that the potential complexity or variability introduced by exon 2 can be reduced by focusing on exon 1 alone as the most critical and immunogenic part of Tat (8). Immunoinformatics approaches with a wide range of tools have greatly advanced vaccine development (6).

## 2. Objectives

In the current study, to the best of our knowledge, we compared for the first time the efficiency of the whole sequence of Tat^(exons 1 + 2)^ with its first exon^(exon 1)^ in a fusion vaccine construct harboring the whole sequence of the Nef protein [i.e., Nef-Tat^(exons 1 + 2)^ and Nef-Tat^(exon 1)^ fusion proteins] using in silico studies.

## 3. Methods

### 3.1. Design of Fusion Protein Constructs

Reference sequences of HIV-1 Nef and Tat proteins were taken from the National Center for Biotechnology Information (NCBI) database (pNL4-3 accession No.: AF324493.2). The whole sequence of the Nef protein was connected to the whole sequence of the Tat protein^(exons 1 + 2)^, and also to the first exon of the Tat protein^(exon 1)^. The SnapGene^®^ 3.2.1 tool was utilized to design the constructs [i.e., Nef-Tat^(exons 1 + 2)^ and Nef-Tat^(exon 1)^].

### 3.2. Prediction of Protein Secondary Structure

The PROTEUS Structure Prediction Server 2.0 (http://www.proteus2.ca/proteus2/) was employed to determine the secondary structural configurations of the fusion constructs using default parameters (9).

### 3.3. Three-Dimensional Modeling of the Protein Constructs

The Robetta server (https://robetta.bakerlab.org) analyzed 3D models of the constructs using specified parameters, and the models showing the highest similarity were selected for further study.

### 3.4. Refinement and Validation of Tertiary Structure

GalaxyRefine (http://galaxy.seoklab.org/cgi-bin/submit.cgi?type=REFINE2) and SAVE6.0 (https://saves.mbi.ucla.edu/) were used to refine and validate the modeled tertiary structures, respectively. The structure stereochemical qualities of the modeled proteins were analyzed by ERRAT, Procheck, and Verify 3D tools (10, 11).

### 3.5. Determining Physicochemical Parameters

ProtParam (https://web.expasy.org/protparam/) assessed the physicochemical properties of the constructs, while ToxinPred (https://webs.iiitd.edu.in/raghava/toxinpred/), AllerTOP (https://www.ddg-pharmfac.net/AllerTOP/), and VaxiJen v2.0 (http://www.ddg-pharmfac.net/vaxijen/VaxiJen/VaxiJen.html) evaluated their toxicity, allergenicity, and antigenicity, respectively.

### 3.6. Protein Disulfide Bonds Prediction

The DIpro server (http://scratch.proteomics.ics.uci.edu/) predicted disulfide bonds and cysteine bonding states using specified parameters (12).

### 3.7. Molecular Docking Analysis of Protein-Receptor

ClusPro (https://cluspro.bu.edu/) and HDOCK servers (http://hdock.phys.hust.edu.cn) were used to identify the optimal orientation and matching pattern with the minimal energy level and the strongest binding affinity between the validated constructs (as ligands) and different TLRs (TLR-2 to TLR-5, and TLR-7 to TLR-9) (13). The HDOCK score indicated several energy terms and measures the quality of the predicted binding mode. A better (more negative) docking score generally indicates a more favorable and potentially stable binding mode (14). The resulting docking data was visualized using ChimeraX-1.1 software. Ligplot software (https://www.ebi.ac.uk/thornton-srv/software/LigPlus/) was used to generate 2D schematic representations of the interactions between TLR-4 and constructs (15).

### 3.8. Immune Simulation

The C-IMMSIM server (https://kraken.iac.rm.cnr.it/C-IMMSIM/) was used to simulate the immune system and predict results of immune response after administrations of the fusion proteins as vaccine constructs (16). Three injections were given at 0, 48, and 90 hours (injections 1, 2, and 3), two weeks apart, using default simulation parameters. Each time step in the simulation corresponded to 8 hours.

### 3.9. Molecular Dynamic Simulation Analysis

We used the AlphaFold Protein Structure Database (https://alphafold.ebi.ac.uk) to obtain the full-length structure of TLR-4, which served as the starting structure for molecular dynamic (MD) simulations. To provide more detailed insight into the dynamic behavior of Nef-Tat^(exons 1 + 2)^ and Nef-Tat^(exon 1)^ constructs in complex with TLR-4, MD simulations were performed using the CHARMM36 force field in the GROMACS 2020.3 package (17). The docked complexes with high binding affinities were subjected to MD simulation for a duration of 100 ns. The complexes were placed in a cubic box, maintaining a uniform edge distance of 10 Å, and placed in a solvent environment using the transferable intermolecular potential 3P (TIP3P) water model. Additionally, the complex system was neutralized by introducing counterions, Na^+^ and Cl^-^ (18).

The solvated system underwent energy minimization utilizing 50,000 steps of steepest descent with a convergence tolerance of 1000 kJ/mol/nm. The equilibration was a two-step process under periodic boundary settings, which involved the NVT (constant volume) and the NPT (constant pressure and temperature) maintaining a pressure of 1 bar and temperature at 300 K (physiological temperature), respectively. Temperature was regulated using a velocity rescaling approach with a 0.1 fs time step, and pressure was managed using the Berendsen barostat with a coupling time of 2 ps (19). The electrostatic interactions were computed using the particle mesh Ewald (PME) algorithm (20) with Coulomb and van der Waals cut-offs set at 1.0 nm. Finally, the MD simulation was performed for 100 ns with a 2 fs time step under conditions of 300 K and 1 bar. Following the simulations, coordinates were recorded in trajectory files every 20 ps for further analysis of structural metrics such as root mean square deviation (RMSD), root mean square fluctuation (RMSF), and radius of gyration (Rg).

### 3.10. In Silico Cloning of the Vaccine Constructs

The Java Codon Adaptation Tool was used to adapt codon usage of the designed vaccines for expression in *Escherichia coli*, measure Codon Adaptation Index (CAI), and GC content. DNA stability, melting temperature (Tm), secondary structure formation, and transcription efficiency are affected by GC content. The SnapGene 3.2.1 Tool was used to insert the codon-optimized sequences into the pET24a (+) vector for bacterial expression (21).

## 4. Results

### 4.1. Protein Secondary and Three-Dimensional Structures

The protein secondary structures of the fusion protein constructs (Appendix 1 in Supplementary File) were predicted (Table 1). The predicted structure by the PROTEUS2 server showed a higher percentage of helix and lower coil contents for the Nef-Tat^(exon 1)^ structure compared to the Nef-Tat^(exons 1 + 2)^ structure (Appendix 2A in Supplementary File). The 3D models with the greatest degree of homology were selected for further analysis (Appendix 2B in Supplementary File).

**Table 1.** Protein Secondary Structure Prediction

Constructs	Overall Confidence Value; %	Predicted Helix Content; % (residues)	Predicted Beta Sheet Content; % (residues)	Predicted Coil Content; % (residues)	Predicted Signal Peptide Content; % (residues)	Predicted Membrane Content; % (residues)
**Nef-Tat** ^**(exons 1** **+** **2)**^	72.6	21 (63)	7 (21)	72 (212)	0 (0)	0 (0)
**Nef-Tat** ^**(exon 1)**^	72.1	29 (82)	9 (25)	62 (174)	0 (0)	0 (0)

### 4.2. Refinement and Model Quality of Tertiary Structures

The refinement process was utilized to identify and minimize potential errors in the 3D structures (Figure 1A and E). Verify3D analysis showed over 80% of residues in both models scored above 0.1, confirming successful validation (Figure 1B and F). The ERRAT server predicted overall quality factors (OQFs) of 82.05 and 92.7 for the Nef-Tat^(exons 1 + 2)^ and Nef-Tat^(exon 1)^ constructs, respectively (Figure 1D and G). 82.7% and 87.4% favored regions were determined on the Ramachandran plot of the 3D validated structures of the Nef-Tat^(exons 1 + 2)^ and Nef-Tat^(exon 1)^ structures, respectively (Figure 1C and H; Appendix 3 in Supplementary File). These results show the models have reliable amino acid conformations and good quality.

**Figure 1.**
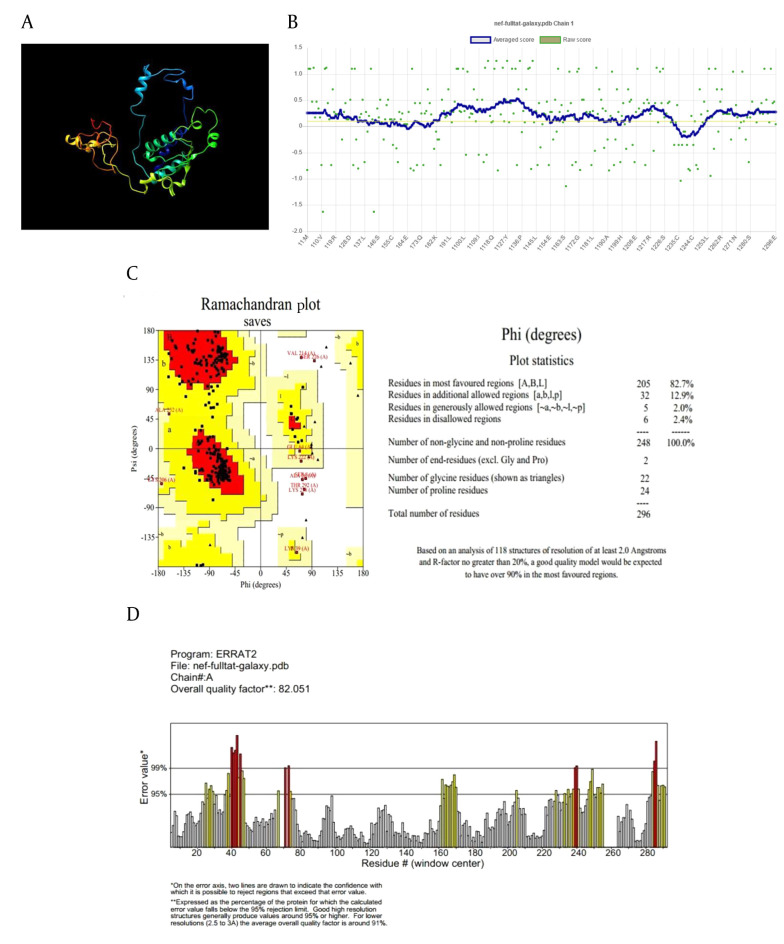
Structural validation of the modeled constructs: Refinement and validation of 3D structures of the A-D, Nef-Tat^(exons 1 + 2)^ and E-H, Nef-Tat^(exon 1)^ construct; A & E, the structural refinement; B & F, verify 3D plot; D & G, ERRAT plot; C & H, Ramachandran plots of the constructs.

**Figure 1.**
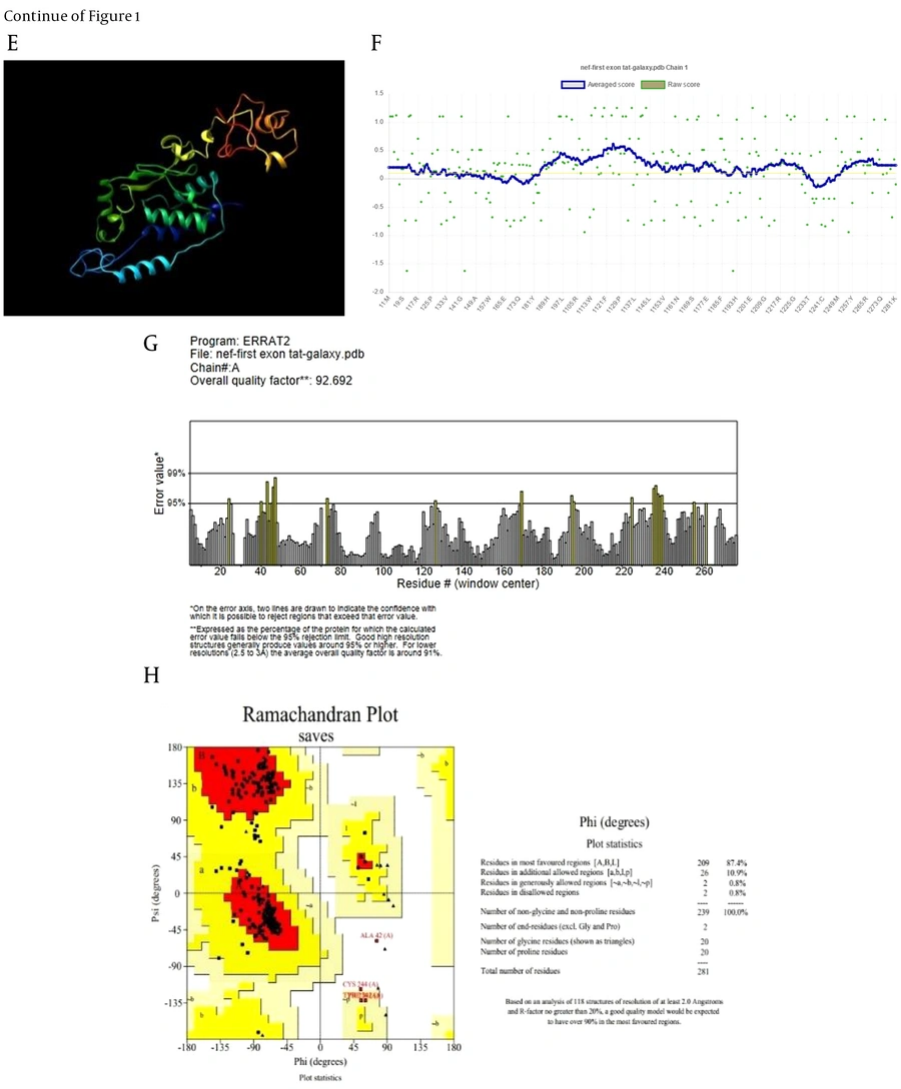
Structural validation of the modeled constructs: Refinement and validation of 3D structures of the A-D, Nef-Tat^(exons 1 + 2)^ and E-H, Nef-Tat^(exon 1)^ construct; A & E, the structural refinement; B & F, verify 3D plot; D & G, ERRAT plot; C & H, Ramachandran plots of the constructs.

### 4.3. Protein Disulfide Bonds and Physicochemical Parameters

The disulfide bond prediction revealed that cysteines at positions 55, 142, 206, 232, 240, 241, 244, and 247 for both Nef-Tat^(exons 1 + 2)^ and Nef-Tat^(exon 1)^ constructs were involved in the creation of four disulfide bonds. Both constructs were non-toxic and non-allergen. The threshold for the antigenicity model is 0.4, thus both constructs were diagnosed as probable antigens. The physicochemical and immunological characteristics of the constructs were shown in Appendix 4 in Supplementary File.

### 4.4. Protein-Receptor Docking

**Figure 2.**
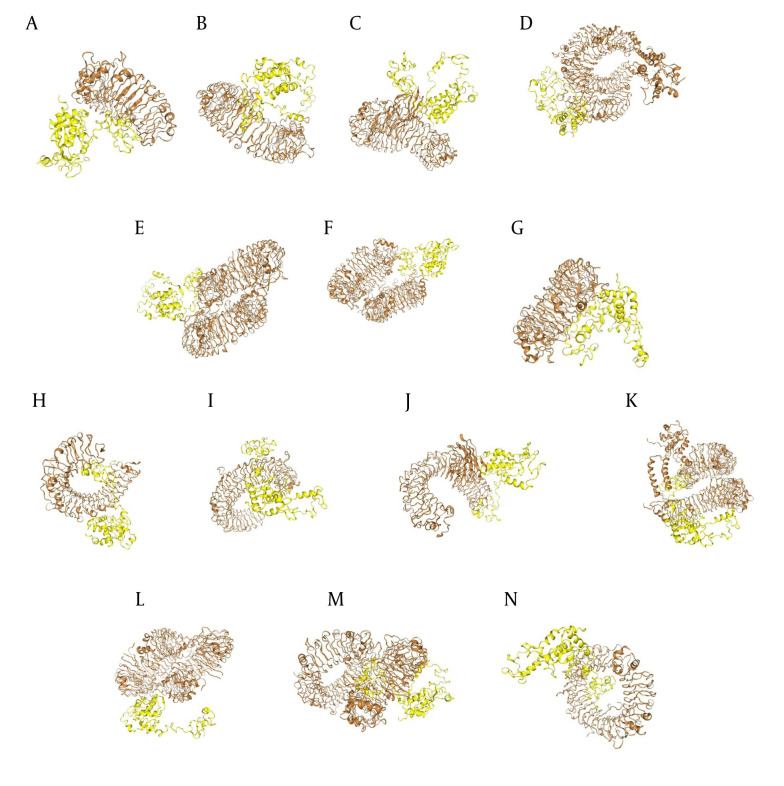
The protein-protein docking between TLRs and the modeled constructs: Docking results between TLRs (brown), the A-G, Nef-Tat^(exons 1 + 2)^ and H-N, Nef-Tat^(exon 1)^ construct; interaction of the Nef-Tat^(exons 1 + 2)^ construct with A & H, TLR-2; B & I, TLR-3; C & J, TLR-4; D & K, TLR-5; E & L, TLR-7; F & M, TLR-8; and G & N, TLR-9.

ClusPro docking analysis revealed that the Nef-Tat^(exons 1 + 2)^ exhibited the strongest binding affinity with TLR-5, while the Nef-Tat^(exon 1)^ showed the highest docking score with TLR-9. Moreover, the Nef-Tat^(exons 1 + 2)^ construct had lower docking score with most of the examined TLRs compared to the Nef-Tat^(exon 1)^ construct (Table 2). Figure 2 shows the best docking model schematics. Due to similar docking results of TLR-4 with both Nef-Tat^(exons 1 + 2)^ and Nef-Tat^(exon 1)^, its inhibitory role in HIV-1 replication, and direct binding of the Tat protein to TLR-4 (22), TLR-4 was chosen to further study its binding to the constructs using Ligplot software. It was demonstrated that both constructs had the same interacted residues, hydrophobic, and hydrogenic bonds for interaction with TLR-4. Therefore, it can be concluded that the second exon of Tat does not play a significant role in the stability of the interactions with TLR-4 (Appendix 5 in Supplementary File).

**Table 2.** Ligand-Receptor Docking Results

Constructs	TLR-2		TLR-3		TLR-4		TLR-5		TLR-7		TLR-8		TLR-9	
	ClusPro	HDOCK	ClusPro	HDOCK	ClusPro	HDOCK	ClusPro	HDOCK	ClusPro	HDOCK	ClusPro	HDOCK	ClusPro	HDOCK
**Nef-Tat** ^**(exons 1** **+** **2)**^	-969.6	-293.94	-977.6	-297.28	-1128.4	-282.53	-1264.7	-301.57	-1031.4	-319.72	-1098.0	-281.21	-1215.2	-387.43
**Nef-Tat** ^**(exon 1)**^	-1021.9	-300.11	-1103.7	-334.27	-1125.0	-309.52	-1425.4	-318.60	-1011.5	-299.69	-1286.4	-350.06	-1476.0	-345.23

### 4.5. Immune Simulation

**Figure 3.**
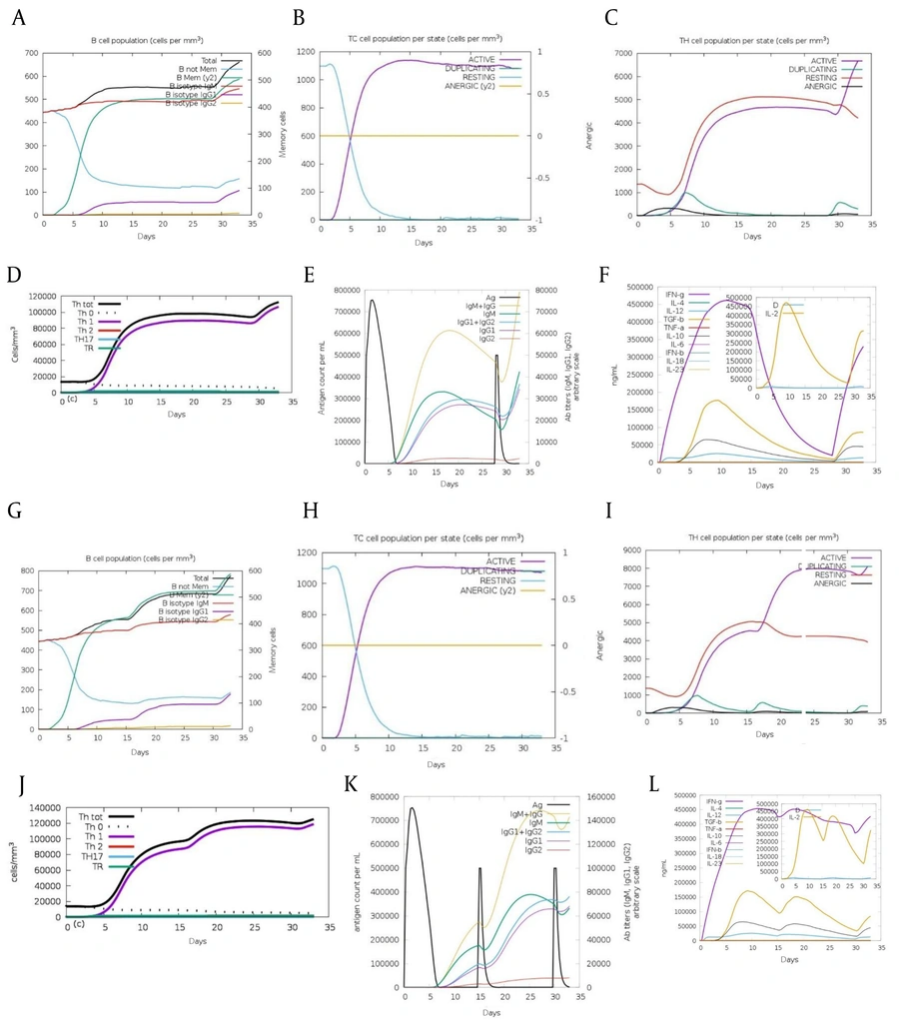
Immune simulation results of the modeled A-F, Nef-Tat^(exons 1 + 2)^ and G-L, Nef-Tat^(exon 1)^ constructs by C-ImmSim: The evolution of A & G, B-cell populations after three injections; B & H, TC-cell populations per state after three injections; C & I, TH-cell populations per state after three injections; D & J, the generation of TH cells; E & K, the production of immunoglobulins; and F & L, the cytokine profile.

The initial immune response to an antigen mainly produces IgM antibodies, with some IgG. IgM levels significantly increased after the first injection of both Nef-Tat^(exons 1 + 2)^ and Nef-Tat^(exon 1)^ proteins, indicating the primary immune response. The secondary immune response, triggered by repeated antigen exposure, showed increased IgM and IgG, especially IgG1, with no significant IgG2 rise for both fusion proteins. Helper T-cells showed a strong response, promoting the development of cytotoxic and Th1 cells. Th1 cell levels stayed consistently high during all exposures to both fusion proteins. Additionally, memory B-cell numbers increased during the administration of both fusion proteins (Figure 3A-E vs. Figure 3G-K). Results showed interferon (IFN)-γ levels were notably higher than other cytokines during immunization with both fusion proteins (Figure 3F vs. Figure 3L). 

### 4.6. Molecular Dynamic Simulation

As previously described, TLR-4 was chosen for further MD study. Complex stability was examined via RMSD. The RMSF measured side chain fluctuations of residues in TLR-4 complexes, while Rg assessed protein compactness during MD simulation. The RMSD plot presented that the fluctuation of the Nef-Tat^(exon 1)^ structure stopped its upward trend after ~35 ns; however, Nef-Tat^(exons 1 + 2)^ showed increasing behavior until ~45 ns and presented a constant pattern of fluctuation up to 100 ns. Moreover, a lower degree of fluctuation within 0.25 - 0.3 nm of RMSD over simulation time was observed, indicating structural integrity and/or firm binding in both complexes [Nef-Tat^(exon 1)^-TLR-4 and Nef-Tat^(exons 1 + 2^)-TLR-4]. The RMSD average values for Nef-Tat^(exon 1)^, Nef-Tat^(exons 1 + 2)^, and TLR-4 were found to be 0.14 (0.08 to 0.2), 0.2 (0.08 to 0.32) nm, and 0.12 (0.1 to 0.15), respectively.

**Figure 4.**
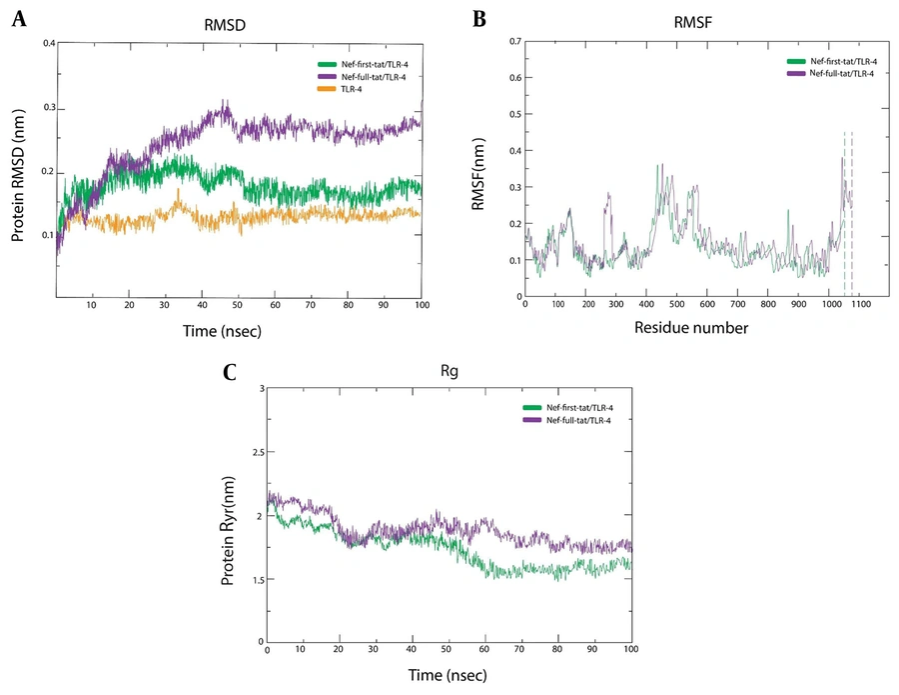
Molecular dynamic (MD) simulation of the Nef-Tat^(exon 1)^ and Nef-Tat^(exons 1 + 2)^ constructs/TLR-4 complex: A, RMSD plot; B, RMSF plot; and C, Rg plotted against simulation time.

All RMSD averages, especially for Nef-Tat^(exon 1)^, were below 0.2 nm, confirming reliable MD simulation equilibrium (Figure 4A). The high value of RMSF and low value of RMSF showed the flexible region and limited movements, respectively. The RMSF profile revealed a fluctuation value of less than 2Å for all residues, which may indicate low variations in the Nef-Tat^(exon 1)^ and Nef-Tat^(exons 1 + 2)^ structures. Further to the N- and C-terminal residues, residual fluctuations were present in other regions of both Nef-Tat^(exon 1)^ and Nef-Tat^(exons 1 + 2)^ structures, including the regions around 125 to 150 and residues of 283, 287, 291, 294 in the Nef-Tat^(exons 1 + 2)^ sequence, due to the presence of a Proline residue. The Nef-Tat^(exons 1 + 2)^ construct showed the biggest dynamic fluctuations around 283 to 294. The RMSF average values for Nef-Tat^(exon 1)^ and Nef-Tat^(exons 1 + 2)^ were 0.175 (0.05 to 0.3) and 0.2 (0.05 to 0.35), respectively (Figure 4B). 

Additionally, high and low Rg values during MD simulations demonstrated less and greater compactness of protein structure, respectively. The average values of Rg for Nef-Tat^(exon 1)^ and Nef-Tat^(exons 1 + 2)^ with TLR-4 were 1.74 and 1.90, respectively. The Rg plots of the protein constructs showed fluctuations ranging less than 2Å, suggesting that the protein constructs were stable. The Rg results align well with RMSD and RMSF (Figure 4C). 

### 4.7. In Silico Cloning of the Constructs

A CAI of 1.0 indicated optimal codon usage and high expression potential of both constructs in *E. coli*. GC content of 50.73% reflected sequence stability and suitability for the host. The codon-modified sequences were inserted into the pET24a (+) vector for bacterial expression (Appendix 6 in Supplementary File).

## 5. Discussion

Selecting the most suitable immunogen is crucial for developing an efficacious HIV-1 vaccine (23). Preclinical studies demonstrated that Tat is safe and elicits a specific immune response (7). Some phase I (ISS T-001, NCT00505401) (24) and II (ISS T-002, NCT00751595) (25) therapeutic trials were conducted based on the Tat protein. On the other hand, Nef-specific T-cell immunity is essential to control HIV-1 viral load, and it is highly conserved and immunogenic for vaccine development (26). HVTN 505, HVTN 502, 503, 111, and IAVI are some clinical trials focused on Nef (27). Many studies used an immunoinformatics approach to develop vaccines against infectious diseases such as HCV (28) and HIV (6).

In this study, we compared the immune properties of the first exon of the Tat protein^(exon 1)^ and the whole sequence of the Tat protein^(exons 1 + 2)^ as linked to the whole sequence of Nef using in silico studies for the first time. It is important to determine whether exon 2 is necessary for increasing the potency of the vaccine construct. Our findings showed that deletion of exon 2 within the fusion construct results in minor changes to the secondary structures. Notably, the helix content of the Nef-Tat^(exon 1)^ construct was higher than the Nef-Tat^(exons 1 + 2)^ construct, which can be a reason for its greater stability (29). Both constructs were non-allergenic and non-toxic, but the Nef-Tat^(exon 1)^ construct had higher antigenicity. Both constructs had a molecular weight of < 70 kDa, which is ideal for a vaccine particle (30); however, the Nef-Tat^(exon 1)^ construct had a lower molecular weight (32 kDa) than the Nef-Tat^(exons 1 + 2)^ construct. In our other study, we also found that the Nef-Tat fusion protein consisting of the first domain of Tat and the full length of the Nef antigen induced higher levels of IgG2a, IFN-γ, and granzyme B compared to the Nef antigen in BALB/c mice, notably when applied in a heterologous prime/boost regimen (31).

Predicting disulfide connectivity is key to understanding protein folding, stability, structure, and function (32). The constructs were predicted to have the same number of disulfide bonds, indicating their stability and consistent structural organization. In this study, structural models were created by the Robetta server, and then refinement was processed by the SAVE6.0 web server. The accuracy and validity of all protein models were confirmed.

The interactions of fusion constructs with seven TLRs were investigated using the ClusPro and HDOCK servers, and their results confirmed each other. Seven TLRs, including TLR-2 to TLR-5 and TLR-7 to TLR-9, which are key innate immune receptors that recognize HIV or viral components and are involved in Th1 or Th2 activity, were evaluated in the docking analysis. TLR-2 is the most powerful receptor and recognizes a wide variety of PAMPs (33). TLR-3 and TLR-5 have shown strong binding affinity with HIV vaccine constructs (34). Many studies reported that HIV is directly identified by TLR-4 (35). TLR-8 agonists activate DCs and boost Th1 and CD8+ T-cell responses, enhancing vaccine efficacy (36). Activation of TLR-7 inhibits HIV viral production and intensifies the antiviral responses (37). It was demonstrated that the stimulation of TLR-9 in plasmacytoid DCs boosts the generation of type I IFNs, providing protection against HIV infection (38). Conversely, an in vitro study demonstrated that activation of TLR-9 can reactivate latent HIV in CD4+ T-cells (39). Activation of TLR-5 was shown to enhance HIV transcription in HIV-infected central memory CD4+ T-cells (40). Thus, TLR-5-targeting vaccines may worsen HIV-1 by activating CD4+ T-cells and reactivating latent proviruses (40). Altogether, analyzing the interactions of HIV vaccine targets with TLRs using in silico studies is crucial for developing effective vaccines.

Our data demonstrated that both constructs could interact with the TLRs. In most cases, better docking results were observed for the Nef-Tat^(exon 1)^ construct. Among TLRs, TLR-4 showed high and similar docking scores for both constructs. Furthermore, the Tat protein directly binds to TLR-4 and activates the production of tumor necrosis factor-α (TNF-α) and interleukin-10 (IL-10) (22). Thus, the TLR-4/protein complexes were used for MD simulation. In agreement with the method used in our study, a recent study subjected the best-docked complexes of vaccine constructs with TLRs (TLR-5 between TLR-2 to TLR-5) to MD simulation (41). Consistent outcomes were obtained from the RMSD, RMSF, and Rg results, demonstrating that the conformational models were stable. Our results showed that the Nef-Tat^(exon 1)^/TLR-4 complex was more stable and had more limited movements compared to the Nef-Tat^(exons 1 + 2)^/TLR-4 complex. In this regard, the elimination of the second exon of Tat^(exon 2)^ indicated better results in MDs.

In silico cytokine analysis may indicate low-level production of IL-10 after the second injections of both constructs. The production of IFN-γ and IL-2 was more stable in the Nef-Tat^(exon 1)^ regimen. We observed a sharp drop in IFN-γ production in the Nef-Tat^(exons 1 + 2)^ construct at the threshold of the third injection, while this drop was not observed in the Nef-Tat^(exon 1)^ construct. These results demonstrated that the Nef-Tat^(exon 1)^ regimen can potentially guide the immunity towards Th1 cellular immunity. Indeed, a Th1 immune response was seen in both fusion proteins; but after the second injection, a better Th1 immune response was observed in the Nef-Tat^(exon 1)^ construct.

Both constructs can activate T- and B-cells after primary and subsequent doses. They showed the same active cytotoxic T-cells population and a similar increase in IgM levels during the initial injection. In the first injection, we observed a greater increase of IgM+IgG in the Nef-Tat ^(exons 1 + 2)^ construct. In contrast, in the second and third dose administration, a greater enhancement of IgM+IgG was observed in the Nef-Tat^(exon 1)^ construct.

In a recent study, all protein sequences of HIV-1a and HIV-1b were in silico cloned (42). Our results of in silico cloning indicated both vaccine constructs have the same high potential for expression in the host, and the deletion of the second exon of Tat has no effect on cloning efficacy. The results of structural, physicochemical, and immunological predictions for both protein constructs showed higher potency of the Nef-Tat^(exon 1)^ construct as a vaccine candidate compared to the Nef-Tat^(exons 1 + 2)^ construct. Although our previous study demonstrated the immunogenicity of Nef-Tat^(exon 1)^ (31), in vitro and in vivo experiments are highly required to compare the immunogenicity of Nef-Tat ^(exon 1)^ and Nef-Tat^(exons 1 + 2)^, which can circumvent the limitation of this in silico experiment.

### 5.1. Conclusions

Our results indicated that the Nef-Tat^(exon 1)^ construct could strongly activate both T- and B-cells, and inclusion of the second exon of Tat could not significantly enhance the immunogenicity of the Nef protein. The higher secretion of IL-2 and IFN-γ cytokines (i.e., tendency towards Th1 immunity) was detected for the Nef-Tat^(exon 1)^ construct compared to the Nef-Tat^(exons 1 + 2)^ construct. Our results suggested the idea of using only the first exon of Tat instead of exons 1+2 in HIV vaccine development.

ijpr-24-1-162036-s001.pdf

## Data Availability

All data are available in the manuscript.
