# Supplementary material for: In Silico Analysis of Tat Exons to Increase the Efficacy of a Nef-Tat-based HIV-1 Vaccine Candidate
Source: Iran J Pharm Res. 2025 Sep 13;24(1):e162036. doi: 10.5812/ijpr-162036 (PMC12523967; doi:10.5812/ijpr-162036)
Supplement: ijpr-24-1-162036-s001.pdf [file ijpr-24-1-162036-s001.pdf]

Supplementary Files

Appendix 1 Supplementary File: The Fusion protein constructs sequences

construct	Protein sequence
Nef-Tat (exons 1+2)	MGGKWSKSSV IGWPAVRERM RRAEPAADGV GAVSRDLEKH GAITSSNTAA NNAACAWLEA QEEEEVGFPV TPQVPLRPMT YKAAVDLSHF LKEKGGLEGLIHSQRRQDIL DLWIYHTQGY FPDWQNYTPG PGVRYPLTFG WCYKLVPVEPDKVEEANKGE NTSLLHPVSL HGMDDPEREV LEWRFDSRLA FHHVARELHPEYFKNCLEGS MEPVDPRLEP WKHPGSQPKT ACTNCYCKKC CFHCQVCFMTKALGISYGRK KRRQRRRAHQ NSQTHQASLS KQPTSQPRGDPTGPKE
Nef- Tat (exon 1)	MGGKWSKSSV IGWPAVRERM RRAEPAADGV GAVSRDLEKH GAITSSNTAANNAACAWLEA QEEEEVGFPV TPQVPLRPMT YKAAVDLSHF LKEKGGLEGLIHSQRRQDIL DLWIYHTQGY FPDWQNYTPG PGVRYPLTFG WCYKLVPVEPDKVEEANKGE NTSLLHPVSL HGMDDPEREV LEWRFDSRLA FHHVARELHPEYFKNCLEGS MEPVDPRLEP WKHPGSQPKT ACTNCYCKKC CFHCQVCFMTKALGISYGRK KRRQRRRAHQ NSQTHQASLS K

Appendix 2 Supplementary File: Prediction of protein A) secondary and B) 3D structures. 1) Nef-Tat (exons 1+2), 2) Nef-Tat (exon 1).

A)

1)

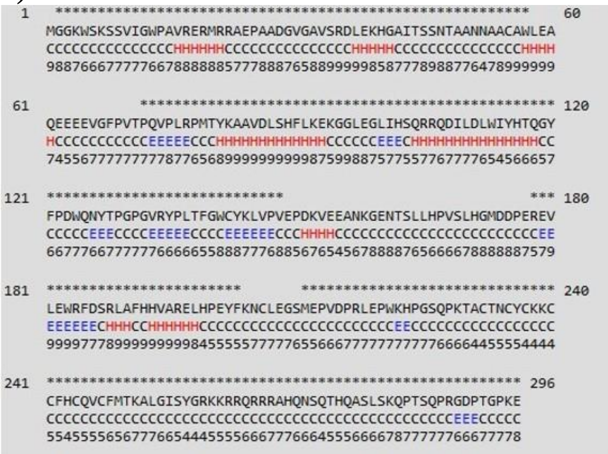

2)

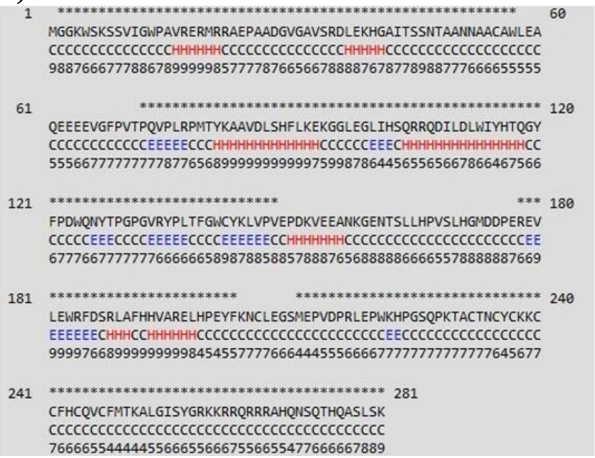

B)

1)

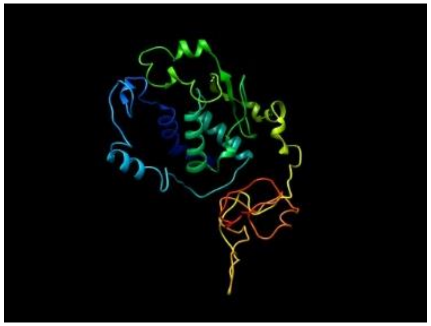

2)

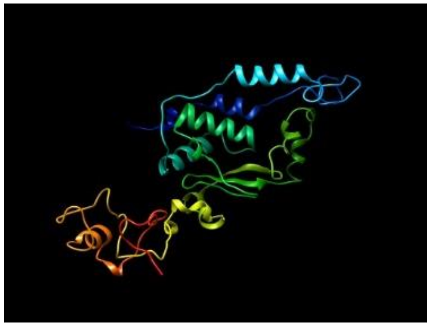

Appendix 3 Supplementary File**:** The structural refinement of the of Nef-Tat (exons 1+2) and Nef-Tat (exon 1) using Ramachandran and ERRAT2 Plot

**Constructs**	**Ramachandran Plot**						**ERRAT2**
	Residues in most favored regions	Residues in disallowed regions	Number of glycine residues	Number of proline residues	Total number of residues	Quality model	Overall quality factor
Nef-Tat (exons 1+2)	82.7%	2.4%	22	24	296	Good	82.051
Nef-Tat (exon 1)	87.4%	0.8%	20	20	281	Good	92.692

Appendix 4 Supplementary File: The physicochemical and immunological characteristics of constructs

**Construct**	**Molecular weight (kDa)**	**pI**	**Negative charge residue**	**Positive charge residue**	**Solubility**	**Allergenicity**	**Antigenicity**
Nef-Tat (exons 1+2)	33.6 kDa	8.62	35	40	Soluble	Non-allergen	0.61
Nef- Tat (exon 1)	32 kDa	8.62	33	38	Soluble	Non-allergen	0.62

Appendix 5 Supplementary File: Interactions (hydrogen and other non-covalent bonds) between TLR-4 and A) Nef-Tat (exons 1+2), B) Nef-Tat (exon 1) using ligplot software.

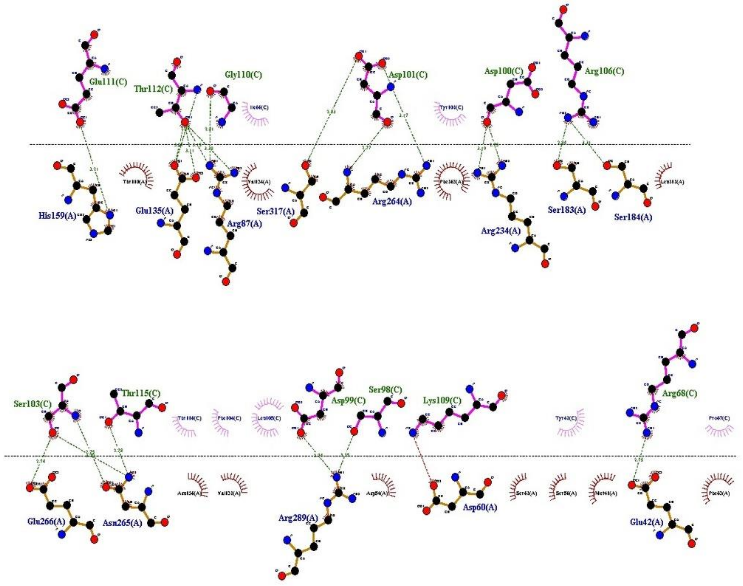

A) nef-full-tat-TLR4

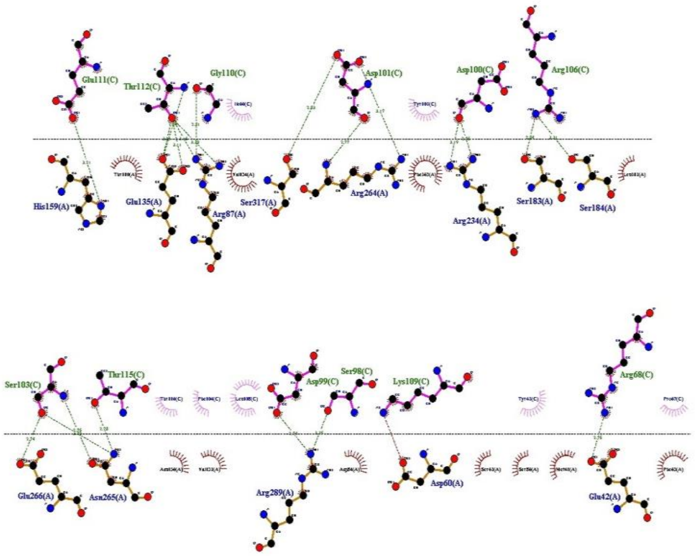

B) nef-first-tat-TLR4

Appendix 6 Supplementary File: *In silico* cloning of vaccine constructs into pET24a (+) prokaryotic expression vector. A) Nef-Tat (exons 1+2) and B) Nef-Tat (exon 1) cloned into pET24a (+) vector by *Sal*I/ *Nhe*I restriction enzymes and marked in blue and red, respectively.

A)

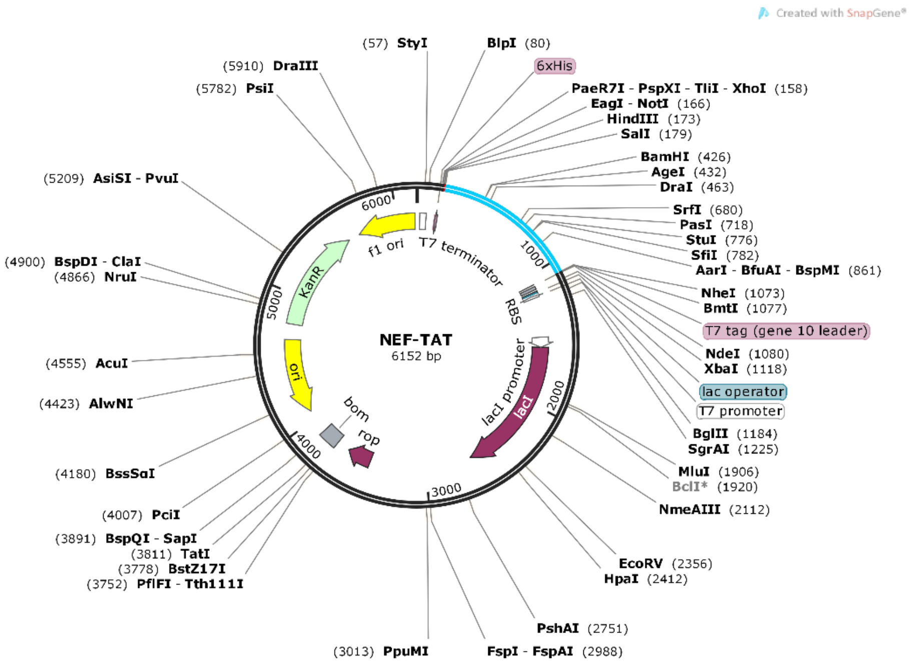

B)

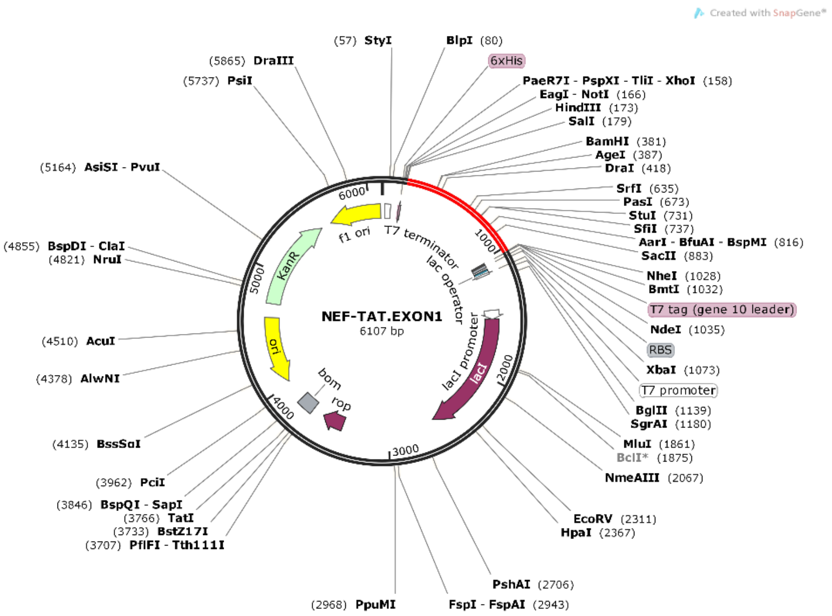
Created with SnapGene®
NEF-TAT.EXON1
6107 bp
(57) StyI
BlpI (80)
6xHis
(5865) DraIII
(5737) PsiI
PaeR7I - PspXI - TliI - XhoI (158)
EagI - NotI (166)
HindIII (173)
SalI (179)
BamHI (381)
AgeI (387)
DraI (418)
(5164) AsiSI - PvuI
SrfI (635)
PasI (673)
StuI (731)
SfiI (737)
AarI - BfuAI - BspMI (816)
SacII (883)
(4855) BspDI - ClaI
(4821) NruI
NheI (1028)
BmtI (1032)
T7 tag (gene 10 leader)
NdeI (1035)
RBS
XbaI (1073)
T7 promoter
BglII (1139)
SgrAI (1180)
MluI (1861)
BclI* (1875)
NmeAIII (2067)
(4510) AcuI
(4378) AlwNI
(4135) BssSαI
(3962) PciI
(3846) BspQI - SapI
(3766) TatI
(3733) BstZ17I
(3707) PflFI - Tth111I
EcoRV (2311)
HpaI (2367)
PshAI (2706)
(2968) PpuMI
FspI - FspAI (2943)
f1 ori
T7 terminator
lac operator
lacI promoter
lacI
KanR
ori
bom
rop
1000
2000
3000
4000
5000
6000
